# rPagSP02+rPagSP06 recombinant salivary antigen is a reliable biomarker for evaluating exposure to Phlebotomus argentipes in Sri Lanka

**DOI:** 10.21203/rs.3.rs-4633976/v1

**Published:** 2024-07-19

**Authors:** Sachee Bhanu Piyasiri, Sanath Senanayake, Nilakshi Samaranayake, Serena Doh, Eva Iniguez, Shaden Kamhawi, Nadira Darshani Karunaweera

**Affiliations:** Department of Parasitology, Faculty of Medicine, University of Colombo, Colombo, 0800, Sri Lanka; Department of Parasitology, Faculty of Medicine, University of Colombo, Colombo, 0800, Sri Lanka; Department of Parasitology, Faculty of Medicine, University of Colombo, Colombo, 0800, Sri Lanka; NIAID, National Institutes of Health; NIAID, National Institutes of Health; NIAID, National Institutes of Health; Department of Parasitology, Faculty of Medicine, University of Colombo, Colombo, 0800, Sri Lanka

**Keywords:** cutaneous leishmaniasis, Phlebotomus argentipes, salivary antigen, vector exposure, surveillance, Sri Lanka

## Abstract

*Phlebotomus argentipes* is the established vector of leishmaniasis in the Indian sub-continent. Antibodies to sand fly salivary antigens are biomarkers for vector-host exposure in leishmaniasis-endemic regions. *Ph. argentipes* transmits *Leishmania donovani* in Sri Lanka, primarily causing cutaneous leishmaniasis (CL). Our study compared the performance of salivary gland homogenate (SGH) from a lab-reared local strain of *Ph. argentipes* females to a composite recombinant salivary biomarker (rPagSP02 + rPagSP06) in a CL-endemic population. Sera from 546 healthy individuals, 30 CL patients, and 15 non-endemic individuals were collected. Western blot analysis of *Ph*. *argentipes* SGH identified immunogenic bands between 15 kDa and 67 kDa, with bands of predicted molecular weight õf 15 kDa (SP02) and ~28–30 kDa (SP06) as the major antibody targets. Indirect ELISAs using SGH or rPagSP02 + rPagSP06 antigens showed high sensitivity (96.7%) and specificity (100%), detecting comparable seropositivity in endemic populations. rPagSP02 + rPagSP06 exhibited enhanced discriminatory ability, supported by a strong positive correlation (r = 0.869) with SGH. Our findings indicate that the composite rPagSP02 + rPagSP06 salivary biomarker effectively identifies *Ph. argentipes* exposure in individuals living in Sri Lanka, showing promising potential for use in surveillance. These findings should be further validated to confirm the epidemiological applications in leishmaniasis-endemic regions.

## Introduction

Leishmaniasis, a neglected vector-borne disease, poses significant public health challenges worldwide [1, 2]. A total of 102 countries and 5 continents have reported endemic leishmaniasis transmission [2]. The disease is caused by *Leishmania* protozoan species and is transmitted by phlebotomine sand flies [3]. *Phlebotomus argentipes* is vector of cutaneous leishmaniasis (CL) and visceral leishmaniasis (VL) in the Indian subcontinent (ISC), with *Leishmania donovani* as the causative agent [4–6].

The World Health Organization (WHO) has set long-term targets for the global elimination of neglected tropical diseases (NTDs), with VL being a primary focus in their 2030 roadmap for NTD eradication. As part of integrated approaches, 56 countries aimed to eliminate VL by 2025, and 64 countries by 2030 [7–9]. However, despite the significant reduction in VL incidence in India, Nepal, and Bangladesh through elimination programs in endemic areas, new cases of CL caused by *L. donovani* have emerged primarily in non-endemic regions [10–16]. Persistence of reservoirs due to atypical *Leishmania* species-phenotype associations pose a challenge for achieving the elimination targets. Sri Lanka was the first country to report CL due to *L. donovani* in the ISC [17, 18], with similar subsequent reports from several other countries, the most recent being from India [19–21]. The elimination initiative has not yet outlined the tools for assessing and maintaining its success, apart from the use of clinical outcomes to measure progress [16].

The salivary proteins of sand flies represent promising biomarkers that induce a specific antibody response in both humans and animals [22]. These proteins hold significant potential as a tool for quantifying exposure to sand fly bites and the risk of contracting leishmaniasis [23–27]. Studies conducted in India, Nepal and Bangladesh demonstrated the use of anti-salivary antibodies to estimate human exposure to *Ph. argentipes* bites [28–32]. A combination of two recombinant *Ph. argentipes* salivary proteins, rPagSP02 + rPagSP06, was recently reported as a reproducibly sensitive and specific biomarker to measure human-vector exposure in a VL endemic population from India [32].

Here, we sought to test the immunogenicity and applicability of the composite salivary biomarker (rPagSP02 + rPagSP06), based on Indian *Ph. argentipes*, as a reliable marker of vector exposure in an endemic population in Sri Lanka.

## Results

### Study population

The characteristics of the study population including living conditions are shown in Table 1. The study participants belonged to varying age groups, with a mean age of 49 ± 15 years. The majority of participants were females (n = 361/546, 66.0%). Occupationally, the participants represented a range of sectors, with the largest group being farmers (n = 92/546, 16.8%). Among the participants, 29.1% (n = 161/546) reported traveling outside their local area within the past 6 months. Housing characteristics revealed that most houses had walls constructed of bricks only (n = 378/546, 69.2%) and tiled roofs (n = 372/546, 68.2%). The participants primarily slept on beds (n = 523/546, 95.8%), and a significant proportion used mosquito nets (n = 407/546, 74.5%). It was notable that 46.0% (n = 251/546) of participants reported using insect repellents. A high proportion (n = 383/546, 70.1%) of participants engaged in outdoor activities during daytime, whereas only a very small minority were active during dusk (14.1%) or dawn (10.8%) when the likelihood of being bitten by sand flies is higher. The presence of cats and dogs or both in the vicinity was reported by 54.6% (n = 298/546) of participants. Regarding the surroundings, the presence of outdoor latrines, store rooms, and cattle sheds was common (n = 338/546, 61.8%) (Table 1).

### Identification of immunogenic antigens in saliva of the local *Ph. argentipes* strain

**Figure 1:**
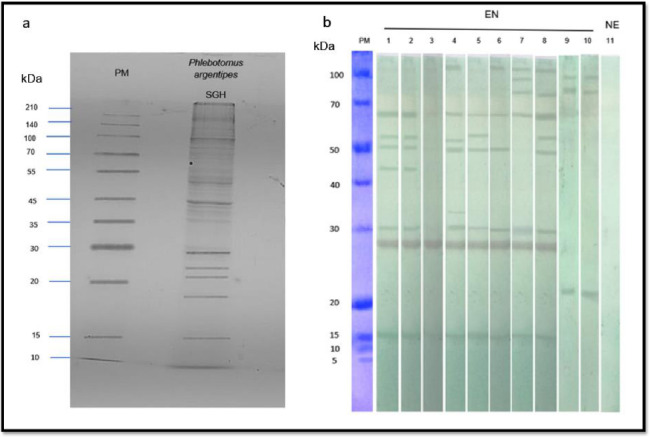
Identification of antigenic *Phlebotomus argentipes* salivary proteins. (**a**) The SGH protein profile of *Ph. argentipes*. 12% SDS–PAGE gel stained by Coomassie Blue. (**b**) Western blots showing the reactivity of IgG in endemic sera against *Ph. argentipes* salivary gland antigens (lane 1–8), or *Culex* spp. salivary gland antigens (lane 9–10: against sera of the endemic individuals screened in lanes 1 and 8). Lane 11: a non-endemic serum sample screened against *Ph. argentipes* salivary gland antigens. PM: protein marker; SGH: salivary gland homogenate; kDa: Kilodaltons; EN: endemic sera; NE: Non-endemic sera. The uncropped gel image and blot image can be found as supplementary Fig. S1 and Fig. S2 online.

SDS-PAGE revealed a diverse range of protein bands for the *Ph. argentipes* SGH of field-collected females from Sri Lanka, with molecular weights ranging from ~10 kDa to ~140 kDa (Fig. 1a). Western blot analysis of serum samples from individuals from a CL endemic region in Sri Lanka had a strong reactivity against *Ph*. *argentipes* SGH, identifying multiple immunogenic bands at approximately ~15 kDa, ~28 kDa, ~30 kDa, ~45 kDa, ~50 kDa, ~55 kDa, and ~67 kDa (Fig. 1b). Notably, the ~28–30 kDa and ~15 kDa bands emerged as major targets of the immune response, along with the ~67 kDa band prominently recognized in seven out of eight endemic individuals (Fig. 1b). Importantly, non-endemic control sera showed no reactivity with SGH of *Ph*. *argentipes* (Fig. 1b).

Crucially, when we tested the sera from subjects 1 and 8, that were strongly reactive against *Ph. argentipes* SGH (Fig. 1b), against SGH of *Culex* spp., mosquitoes that are prevalent in the same area, we did not observe cross-reactivity to *Ph. argentipes* immunodominant proteins, indicating the specificity of the immune response.

### Comparison of anti-saliva antibody responses to SGH and rPagSP02 + rPagSP06

**Figure 2:**
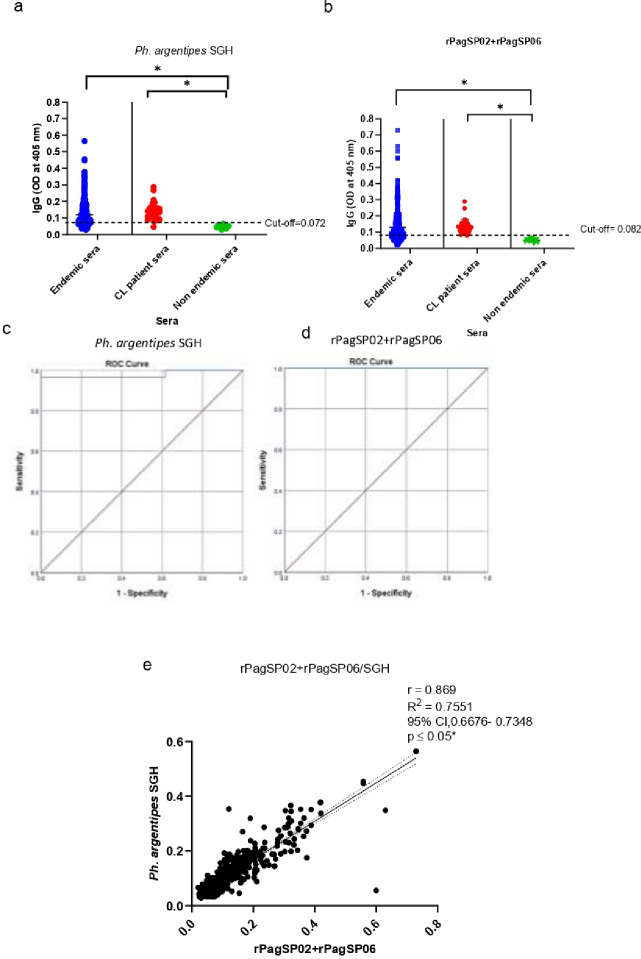
The rPagSP02+rPagSP06 composite biomarker of exposure to *Ph. argentipes* exhibits high sensitivity and specificity in Sri Lanka. (**a**) Anti-salivary IgG antibodies against 2 μg/mL of salivary gland homogenate (SGH) from Sri Lankan *Ph. argentipes*, cut-off at 0.072. (**b**) Anti-salivary IgG antibodies against 1 μg/mL of rPagSP02+rPagSP06 composite recombinant salivary antigen of *Ph. argentipes*, cut-off at 0.082. The cut-off value of the indirect ELISA was calculated using a receiver operating characteristic (ROC) curve with OD values of non-endemic (NE) individuals (negative controls, n=15) and sera from clinically confirmed CL-positive patients (Positive controls, n=30). Antigens were tested against serum samples (1:50 dilution) from 546 individuals living in endemic areas of CL, 30 newly diagnosed CL patients and 15 non-endemic individuals. (**c,d**) Receiver operating characteristic (ROC) curve for indirect ELISA of SGH, area under the curve (AUC) value equals to 0.979 (**c**) and rPagSP02+rPagSP06, AUC value equals to 1.000 (d). (**e**) Pearson’s rank correlation test between *Ph. argentipes* SGH and rPagSP02+rPagSP06. Correlation coefficient r= 0.869 at 95% CI. A two-tailed p < 0.05 as considered statistically significant.

Next, we compared the antibody response to SGH and rPagSP02 + rPagSP06 using indirect ELISA. The antibody levels of healthy and CL patients from the endemic area were significantly higher compared to non-endemic controls for both SGH and rPagSP02 + rPagSP06 (Fig. 2a,b). The cut-off values determined based on the receiver operating characteristic (ROC) curve for these antigens were 0.072 and 0.082, respectively (Fig. 2a,b). The ROC curve analysis showed a sensitivity of 96.7% and specificity of 100% for both antigens (Fig. 2c,d). The rPagSP02 + rPagSP06 antigen had a discriminatory ability, with an area under the curve (AUC) value of 1.000 in the ROC analysis. The SGH antigen also exhibited a high discriminatory power, with an AUC value of 0.979. Furthermore, a strong positive correlation (r = 0.869, 95% CI) was observed between SGH and rPagSP02 + rPagSP06, as determined by Pearson’s rank correlation test (Fig. 2e). In the endemic population consisting of healthy individuals and CL patients (n = 546), both antigens exhibited a considerable proportion of seropositive individuals, with a marginal increase in seropositivity rates observed for the rPagSP02 + rPagSP06 antigen (65.2% versus 63.2% for SGH) (Table 2). The mean absorbance values corresponding to the mean level of anti-saliva IgG antibodies also followed a similar pattern, with moderately higher values for the rPagSP02 + rPagSP06 antigen (0.130 ± 0.090 versus 0.119 ± 0.072 for SGH). Among the CL patients’ sera (n = 30), both antigens were recognized in 29 individuals and exhibited a high sensitivity and specificity with seropositivity rates of 96.6% (Table 2). The mean absorbance values in the CL patients’ sera were comparable for both rPagSP02 + rPagSP06 and SGH, with moderately higher values for the former (0.136 ± 0.044) (Table 2). The mean levels of anti-saliva IgG antibodies were very low in the control nonendemic group with the antigen combination and SGH giving comparable values (0.048 ± 0.010 for rPagSP02 + rPagSP06 antigen and 0.049 ± 0.010 for SGH) (Table 2)

## Discussion

Salivary proteins secreted by sand flies play crucial roles in facilitating blood feeding by exerting anti-coagulant properties [25, 33–35], while specific proteins have the capability to elicit an immune response. [36–39, 30, 31]. Moreover, these salivary proteins hold the potential to be used in predicting disease prevalence and for estimation of the level of sand fly bites and vector exposure [22, 26, 40, 41]. Developing a highly specific and sensitive biomarker to detect human exposure to *Ph. argentipes* bites is of utmost importance in regions endemic to leishmaniasis in the ISC, particularly as tools for monitoring vector control.

The protein repertoire of *Ph. argentipes* saliva from our study aligns with findings by another report, which identified salivary proteins of *Ph. argentipes*, including a D7-related protein (GenBank ID: ABA12141), an antigen 5-related protein (GenBank ID: ABA12137), and an apyrase (GenBank ID: ABA12135) [38]. Moreover, the authors also discovered three PpSP15-like proteins (GenBank ID: ABA12133, ABA12139, and ABA12134), and a 33 kDa-sized protein of unknown function (GenBank ID: ABA12140), which are comparable in size to proteins detected in our study.

We identified seven immunogenic salivary proteins with molecular weights of approximately 15 kDa, 28 kDa, 30 kDa, 45 kDa, 50 kDa, 55 kDa, and 67 kDa, which are similar to those found in a previous study on the salivary antigens of Indian *Ph. argentipes* [32]. Many of the salivary proteins we identified had previously been characterized via Edman degradation [42]. The protein with the size of approximately 15 kDa exhibits similarity to both PpSP15 of *Ph. papatasi* and PagSP02 from *Ph. argentipes*, while those approximately 28 kDa and 30 kDa are likely D7-related proteins, possibly including PagSP06, and those at approximately 45 kDa could be related to yellow proteins [42]. However, immunogenic proteins of approximately 50 kDa and 55 kDa have not been reported before. PagSP06 from *Ph. argentipes* has a predicted molecular weight of 32 kDa, but forms doublets at around 67 kDa [32]. Of note, both PagSP02 and PagSP06 from *Ph. argentipes* were not recognized by sera from individuals bitten by *Ph. papatasi* [32]. Unlike in India, there are no records of *Ph. papatasi* from Sri Lanka according to past literature [43–48].

A PpSP32-like protein (PagSP06) was considered as a biomarker of *Ph. argentipes* exposure in humans in Bangladesh [30]. A more recent study demonstrated that combining PagSP06 and PagSP02 improves performance of the biomarker, resulting in a noticeable decrease in cross-reactive antibodies [32]. Notably, in our experiments, no cross-reaction was observed with the salivary proteins of *Culex* spp. Given that *Culex* mosquitoes are the most abundant insect species in the study area, the absence of cross-reactivity underscores the species specificity of *Ph. argentipes* salivary antigens.

Salivary proteins from sand flies offer valuable information for assessing vector control strategies and gaining a better understanding of vector dynamics [27, 29]. The findings of this present study demonstrate the utility of anti-salivary IgG antibodies as serological markers for assessing exposure to bites of *Ph. argentipes*, the sand fly vector associated with transmission of CL in Sri Lanka. Similar to India [32], the evaluation of the composite recombinant antigen (rPagSP02 + rPagSP06) using indirect ELISA proved to be as effective as SGH in detecting anti-salivary IgG antibodies in Sri Lanka, providing an alternative that is less labor intensive and more reproducible. Interestingly, the level of exposure and the percentage of individuals testing positive for vector exposure were comparable to, or even higher than, those reported in previous studies conducted in various endemic regions such as Bangladesh in August 2014 and India in November 2019 [30, 32]. Of note, our study was conducted during a period when the sand fly density was high.

In conclusion, the composite rPagSP02 + rPagSP06 antigen proved to be an effective approach for assessing direct human-vector contact specific to *Ph. argentipes* sand flies in Sri Lanka. The composite biomarker demonstrated high discriminatory power, strong correlation with SGH, and a promising diagnostic potential. Further research and validation are warranted to fully explore the clinical and epidemiological applications of these antigens in leishmaniasis. By demonstrating the high sensitivity and specificity of the rPagSP02 + rPagSP06 composite biomarker in Sri Lanka, this study reinforces its validity for use in surveillance studies in the ISC, providing a much-needed tool to assess efficacy of interventions and changes in exposure to *Ph. argentipes* bites post-VL elimination.

## Methods

### Study area

The study area, Ambalantota in the Southern province of Sri Lanka, was chosen due to its endemicity for CL infection. The selection was informed by recent CL prevalence data from the Medical Officer of Health (MOH) unit in Ambalantota for the years 2018, 2019, and 2021 [49]. Areas were classified as endemic or non-endemic based on case incidence data spanning the past 18 years (2001 to 2019). An annual incidence rate of less than 1 case per 1,000 people signified non-endemicity, whereas 10 cases or more per 1,000 people indicated an endemic classification [49].

### Collection of human sera

Sera were collected from healthy individuals aged 18 and above who were naturally exposed to sand fly bites (n = 546) that resided in the Ambalantota MOH area, an endemic CL area. Individuals with a history of CL were excluded from the study. Demographic data and information on living conditions were recorded using a standardized and previously-validated questionnaire during a house-to-house survey, following informed consent. Blood collection, with 3 mL per individual, was carried out on a voluntary basis and the sera obtained were stored at −70°C until further use. Negative control serum samples (n = 15) were obtained from healthy donors from non-endemic areas of CL and CL sera (n = 30) collected in a previous Island wide survey and used as positive controls.

### Salivary gland dissection

*Ph. argentipes* (Sri Lankan strain) female sand flies (aged between 5–7 days), maintained at the insectary facility at the Department of Parasitology, Faculty of Medicine, University of Colombo, Sri Lanka, under controlled conditions of 26°C temperature, 75% humidity, and fed on 30% sucrose solution and *Culex spp*. mosquitoes, collected in cattle-baited net traps, were dissected on a glass slide using fine needles in cold phosphate-buffered saline (PBS) with a pH of 7.4. Their salivary glands (20 gland pairs for sand flies, 10 gland pairs for mosquitoes) were stored in 1.5 mL micro-tubes containing PBS at −70°C.

### Preparation of salivary gland homogenate (SGH)

The dissected salivary glands of *Ph. argentipes* (40 pairs in 40 μL PBS) and salivary glands of *Culex spp*. (20 pairs in 20 μl PBS) were frozen in liquid nitrogen for 2 minutes and then thawed in a hot water bath maintained at 37°C for a minute. After thawing, the sample was centrifuged at 14,000 rpm for 1 minute. The resulting pellet was sonicated for 2 minutes and then centrifuged again at 8,000 rpm for 2 minutes. The supernatant was carefully separated from the pellet.

### Sodium dodecyl sulfate polyacrylamide gel electrophoresis and western blotting

The sodium dodecyl sulfate polyacrylamide gel electrophoresis (SDS-PAGE) and western blotting were conducted according to published protocols [32] to identify immunogenic salivary proteins of *Ph. argentipes*. SGH proteins of *Ph. argentipes* (40 μg per lane) and *Culex spp*. (40 μg per lane) were separated on a 12% SDS-PAGE gel under non-reducing conditions. The separated protein bands were then transferred to a nitrocellulose membrane and cut into strips. These strips were blocked overnight at 4°C in 5% non-fat milk diluted in Tris-buffered saline with 0.05% Tween 20 (TBS-Tw) and subsequently incubated for 3 hours with human sera from 8 endemic individual of the study and 2 non-endemic individuals (diluted 1:80 in TBS-Tw). After washing with TBS-Tw, the strips were incubated with peroxidase-conjugated Goat anti-human IgG antibody (diluted 1:4,000 in TBS-Tween) for 1 hour. The chromogenic reaction was developed using a TMB substrate solution. Molecular weight protein markers were used for estimating the sizes of the protein bands.

### Indirect ELISA to detect anti-saliva IgG antibodies

Flat-bottom 96-well polyvinyl chloride untreated microplates (HiMedia, Cat No. RM1239) were coated with 50 μL of 2 μg/mL of *Ph. argentipes* SGH or 1 μg/mL of composite antigen rPagSP02 + rPagSP06. Briefly, the antigens were diluted in carbonate-bicarbonate buffer, pH 9.6, and the plates were incubated overnight at 4°C. The plates were blocked for 2 hours at room temperature with 200 μL of 20% horse serum prepared in Tris-buffered saline with 0.05% Tween 20 (TBST). Hundred microliters of sera were diluted at 1:50 in TBST with 5% horse serum and incubated for 1 hour at 37°C. A secondary alkaline phosphatase-conjugated goat anti-human IgG (H + L) (Novus Biologicals, CO, USA) was incubated at 1:10,000 for 1 hour at 37°C. Hundred microliters of p-nitrophenyl phosphate liquid substrate (Mabtech, Stockholm, Sweden) were added to all wells and incubated for 1 hour at room temperature, and the optical density (OD) values were recorded at a 405 nm wavelength using an ELISA plate reader (BioTek Epoch, Vermont, USA). The results were expressed as optical density (OD). Duplicate wells were maintained for each sample throughout the assay. The correlation between SGH and composite recombinant salivary antigen was estimated.

### Statistical analysis

Statistical analysis and graphs were conducted using GraphPad Prism 8.0 software. The characteristic features of the study population were analyzed using standard descriptive statistics. Pearson’s rank correlation test was used to determine the p-value and the correlation coefficient (r) at a 95% confidence interval (CI). A p-value less than 0.05 was considered statistically significant.

## Figures and Tables

**Table 1:** Demographic characteristics and living conditions of study population

Characteristic feature/s	Mean ± SD^¶^/Percentage/Ratio Endemic participants n = 546		

Age (mean ± SD^¶^)	49 ± 15 years		

18 to 37 years	139 (25.3%)		
38 to 57 years	228 (41.7%)		
58 to 77 years	172 (31.5%)		
78 to 97 years	8 (1.5%)		

Gender (M: F)	185: 361 (1: 2)		

Marital status			

Married	368 (67.4%)		
	
Unmarried	178 (32.6%)		
	
Other	0		

Education			

No proper education	20 (3.7%)		

Grade 1 to 5	38 (7.0%)		

Grade 6 to 11	322 (59.0%)		

Ordinary level	153 (28.0%)		

Advanced level	10 (1.8%)		

Degree or above	3 (0.5%)		

Occupation			

Farmer	92 (16.8%)		

Self-employed	26 (4.8%)		

Government-sector worker	9 (1.6%)		

Private sector	22 (4.0%)		

Volunteer worker	4 (0.7%)		

Retired	18 (3.3%)		

Non-working or not employed	375 (68.7%)		

Past travel history (within 6 months)			

Yes	161 (29.1%)		

No	385 (70.5%)		

Walls of the house constructed of			

Brick only	378 (69.2%)		

Brick and plaster	161 (29.5%)		

Other	7 (1.3%)		

Roof of the house constructed of			

Tiles	372 (68.2%)		

Asbestos	131 (24.0%)		

Other	43 (7.8%)		

Location of participant’s sleeping area			

On the floor	23 (4.2%)		

On the bed	523 (95.8%)		

Use of insect repellants			

Yes	251 (46.0%)		

No	295 (54.0%)		

Use of mosquito nets			

Yes	407 (74.5%)		

No	139 (25.5%)		

Outdoor activity time	**Working hours**		

	**Less than 1 hour**	**1 to 3 hours**	**More than 3 hours**

4 am to 8 am	28 (5.1%)	31(5.8%)	0

8 am to 4 pm	130 (23.8%)	67 (12.3%)	186 (34.0%)

4 pm to 8 pm	29 (5.3%)	37 (6.8%)	11(2.0%)

8 pm to 4 am	0	0	27 (4.9%)

Presence of domestic and peri domestic animals			

Cats/dogs or both	298 (54.6%)		

Poultry/farm animals	14 (2.6%)		

None	234 (42.8%)		

Presence of other buildings and features within 50m from the house			

Outdoor latrines, store rooms and cattle sheds	338 (61.8%)		

Jungles, shrubs, paddy lands, water bodies	93 (17.1%)		

Dump sites, compost pits	43 (7.9%)		

None	72 (13.2%)		

¶ Standard deviation

**Table 2.** Comparison of the performance of rPagSP02+rPagSP06 and SGH as biomarkers of exposure to *Ph. argentipes*

Sera	rPagSP02 + rPagSP06 antigen ^α^		SGH ^β^	
	Seropositive	Seronegative	Seropositive	Seronegative
Endemic population (n = 546)	356 (65.2%)	190 (34.8%)	345 (63.2%)	201 (36.8%)
Mean absorbance ± SD^¶^	0.130 ± 0.090		0.119 ± 0.072	
CL patients’ sera (n = 30)	29 (96.6%)	1 (3.4%)	29 (96.6%)	1 (3.4%)
Mean absorbance ± SD^¶^	0.136 ± 0.044		0.138 ± 0.052	
Non endemic (n = 15)	0 (0%)	15 (100%)	0 (0%)	15 (100%)
Mean absorbance ± SD^¶^	0.048 ± 0.010		0.049 ± 0.010	

¶ Standard deviation,

α Composite recombinant salivary antigen,

β Prepared from a local strain

## Data Availability

Data supporting the conclusions of this article are included within the article. The datasets used and/or analyzed during the present study are available from the corresponding author upon reasonable request.
